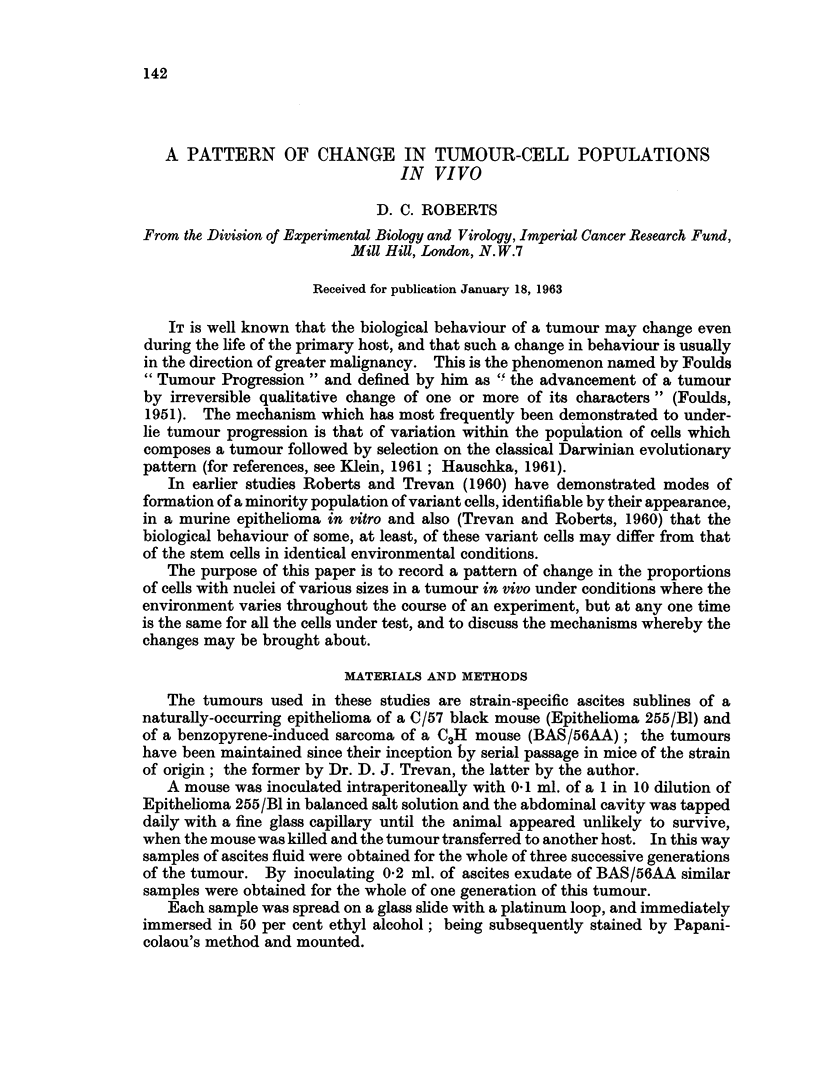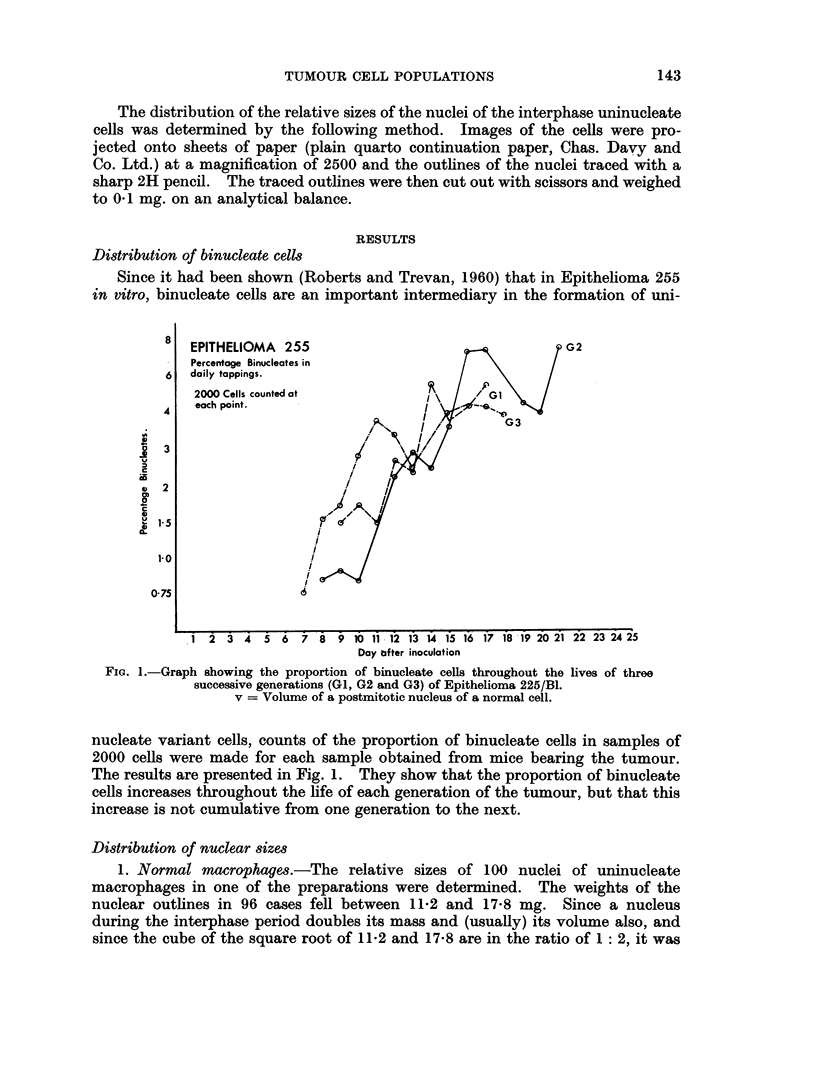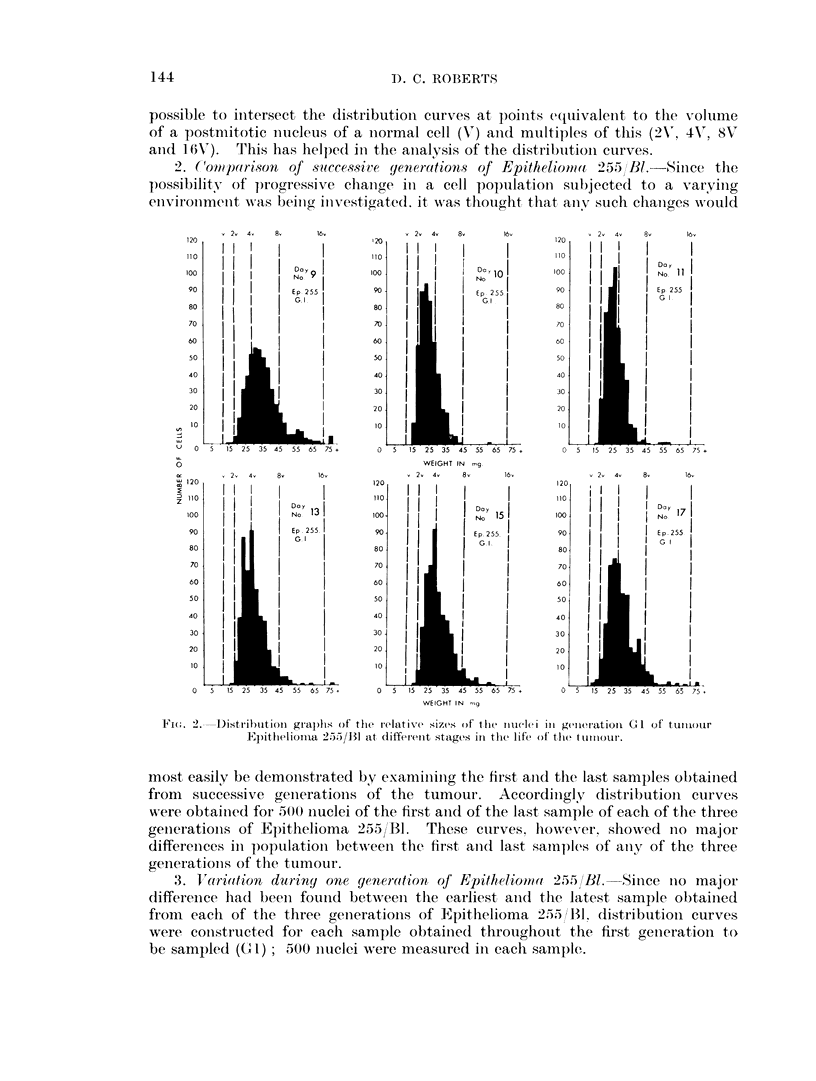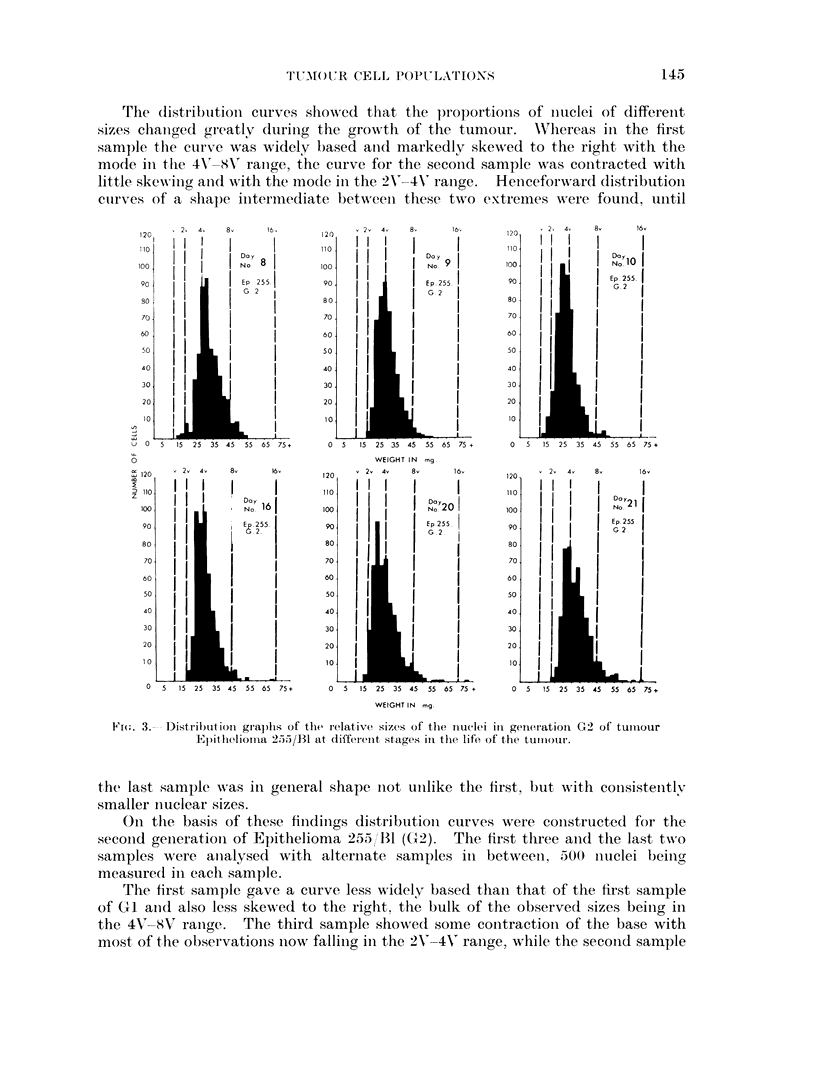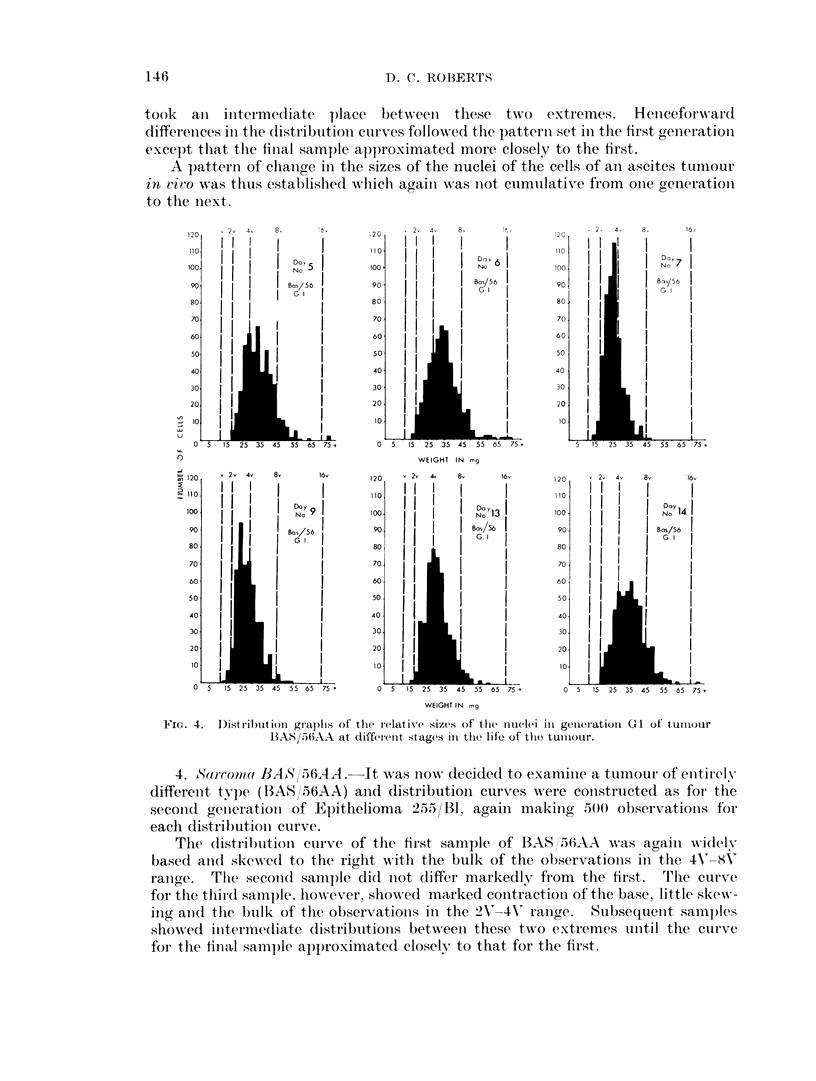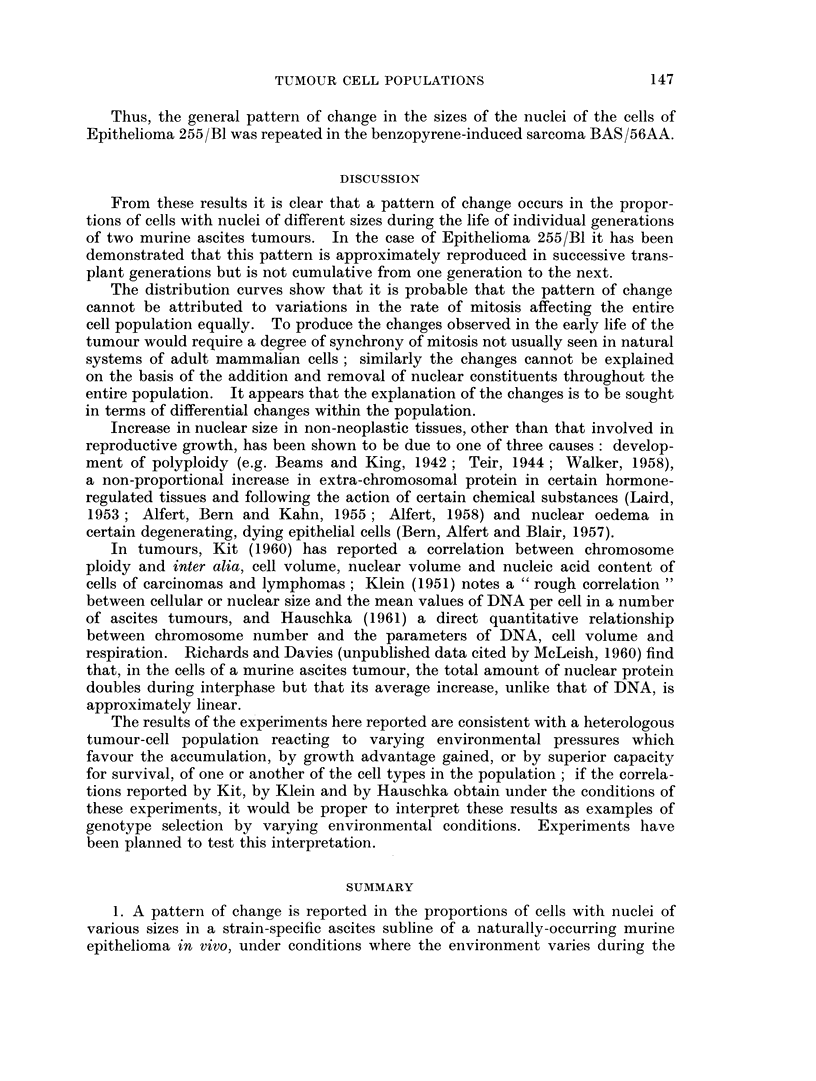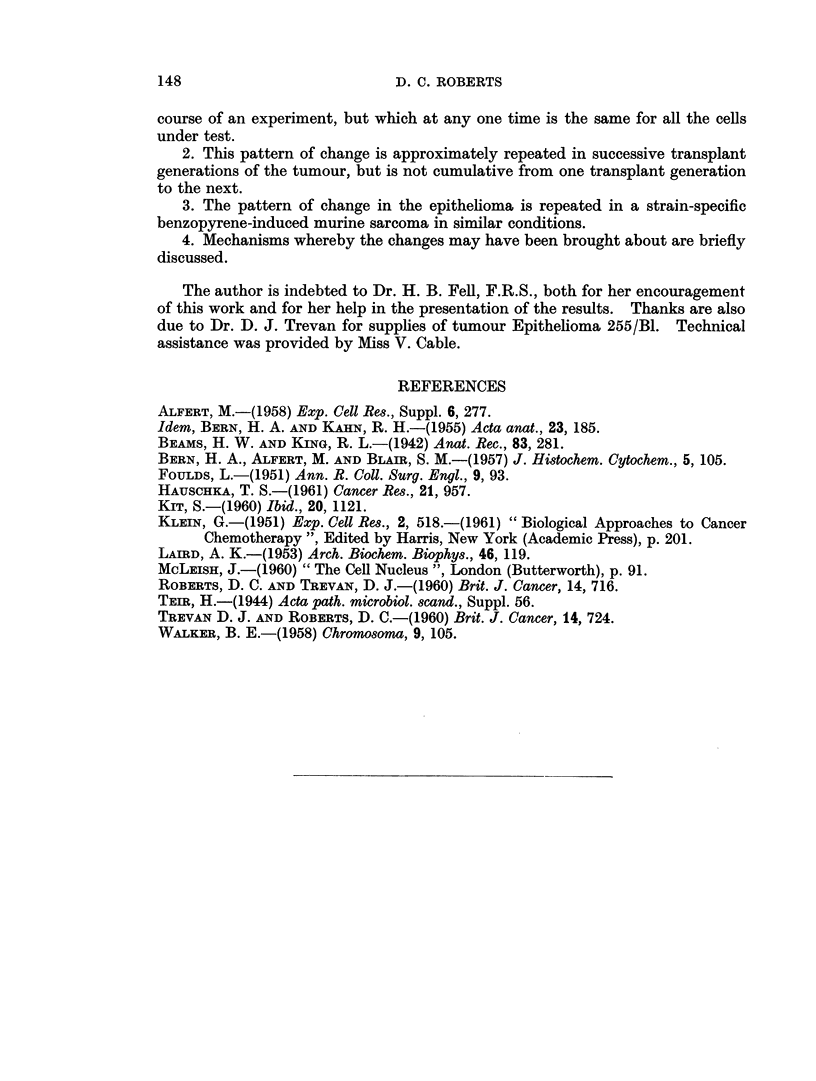# A Pattern of Change in Tumour-cell Populations in vivo

**DOI:** 10.1038/bjc.1963.21

**Published:** 1963-03

**Authors:** D. C. Roberts


### A PATTERN OF CHANGE IN TUMOUR-CELL POPULATIONS *IN VIVO*

D. C. ROBERTS

*From the Division of Experimental Biology and Virology, Imperial Cancer Research Fund, Mill Hill, London, N.W.7*

Received for publication January 18, 1963

IT is well known that the biological behaviour of a tumour may change even during the life of the primary host, and that such a change in behaviour is usually in the direction of greater malignancy. This is the phenomenon named by Foulds "Tumour Progression" and defined by him as "the advancement of a tumour by irreversible qualitative change of one or more of its characters" (Foulds, 1951). The mechanism which has most frequently been demonstrated to underlie tumour progression is that of variation within the population of cells which composes a tumour followed by selection on the classical Darwinian evolutionary pattern (for references, see Klein, 1961; Hauschka, 1961).

In earlier studies Roberts and Trevan (1960) have demonstrated modes of formation of a minority population of variant cells, identifiable by their appearance, in a murine epithelioma *in vitro* and also (Trevan and Roberts, 1960) that the biological behaviour of some, at least, of these variant cells may differ from that of the stem cells in identical environmental conditions.

The purpose of this paper is to record a pattern of change in the proportions of cells with nuclei of various sizes in a tumour *in vivo* under conditions where the environment varies throughout the course of an experiment, but at any one time is the same for all the cells under test, and to discuss the mechanisms whereby the changes may be brought about.

### MATERIALS AND METHODS

The tumours used in these studies are strain-specific ascites sublines of a naturally-occurring epithelioma of a C/57 black mouse (Epithelioma 255/Bl) and of a benzopyrene-induced sarcoma of a $C_3H$ mouse (BAS/56AA); the tumours have been maintained since their inception by serial passage in mice of the strain of origin; the former by Dr. D. J. Trevan, the latter by the author.

A mouse was inoculated intraperitoneally with 0·1 ml. of a 1 in 10 dilution of Epithelioma 255/Bl in balanced salt solution and the abdominal cavity was tapped daily with a fine glass capillary until the animal appeared unlikely to survive, when the mouse was killed and the tumour transferred to another host. In this way samples of ascites fluid were obtained for the whole of three successive generations of the tumour. By inoculating 0·2 ml. of ascites exudate of BAS/56AA similar samples were obtained for the whole of one generation of this tumour.

Each sample was spread on a glass slide with a platinum loop, and immediately immersed in 50 per cent ethyl alcohol; being subsequently stained by Papanicolaou's method and mounted.

The distribution of the relative sizes of the nuclei of the interphase uninucleate cells was determined by the following method. Images of the cells were projected onto sheets of paper (plain quarto continuation paper, Chas. Davy and Co. Ltd.) at a magnification of 2500 and the outlines of the nuclei traced with a sharp 2H pencil. The traced outlines were then cut out with scissors and weighed to 0·1 mg. on an analytical balance.

### RESULTS

*Distribution of binucleate cells*

Since it had been shown (Roberts and Trevan, 1960) that in Epithelioma 255 *in vitro*, binucleate cells are an important intermediary in the formation of uninucleate variant cells, counts of the proportion of binucleate cells in samples of 2000 cells were made for each sample obtained from mice bearing the tumour. The results are presented in Fig. 1. They show that the proportion of binucleate cells increases throughout the life of each generation of the tumour, but that this increase is not cumulative from one generation to the next.

FIG. 1.—Graph showing the proportion of binucleate cells throughout the lives of three successive generations (G1, G2 and G3) of Epithelioma 225/Bl.
v = Volume of a postmitotic nucleus of a normal cell.

*Distribution of nuclear sizes*

1. *Normal macrophages*.—The relative sizes of 100 nuclei of uninucleate macrophages in one of the preparations were determined. The weights of the nuclear outlines in 96 cases fell between 11·2 and 17·8 mg. Since a nucleus during the interphase period doubles its mass and (usually) its volume also, and since the cube of the square root of 11·2 and 17·8 are in the ratio of 1 : 2, it was possible to intersect the distribution curves at points equivalent to the volume of a postmitotic nucleus of a normal cell (V) and multiples of this (2V, 4V, 8V and 16V). This has helped in the analysis of the distribution curves.

2. *Comparison of successive generations of Epithelioma 255/Bl.*—Since the possibility of progressive change in a cell population subjected to a varying environment was being investigated, it was thought that any such changes would

FIG. 2.—Distribution graphs of the relative sizes of the nuclei in generation G1 of tumour Epithelioma 255/Bl at different stages in the life of the tumour.

most easily be demonstrated by examining the first and the last samples obtained from successive generations of the tumour. Accordingly distribution curves were obtained for 500 nuclei of the first and of the last sample of each of the three generations of Epithelioma 255/Bl. These curves, however, showed no major differences in population between the first and last samples of any of the three generations of the tumour.

3. *Variation during one generation of Epithelioma 255/Bl.*—Since no major difference had been found between the earliest and the latest sample obtained from each of the three generations of Epithelioma 255/Bl, distribution curves were constructed for each sample obtained throughout the first generation to be sampled (G1) ; 500 nuclei were measured in each sample.

The distribution curves showed that the proportions of nuclei of different sizes changed greatly during the growth of the tumour. Whereas in the first sample the curve was widely based and markedly skewed to the right with the mode in the 4V–8V range, the curve for the second sample was contracted with little skewing and with the mode in the 2V–4V range. Henceforward distribution curves of a shape intermediate between these two extremes were found, until

FIG. 3.—Distribution graphs of the relative sizes of the nuclei in generation G2 of tumour Epithelioma 255/B1 at different stages in the life of the tumour.

the last sample was in general shape not unlike the first, but with consistently smaller nuclear sizes.

On the basis of these findings distribution curves were constructed for the second generation of Epithelioma 255/B1 (G2). The first three and the last two samples were analysed with alternate samples in between, 500 nuclei being measured in each sample.

The first sample gave a curve less widely based than that of the first sample of G1 and also less skewed to the right, the bulk of the observed sizes being in the 4V–8V range. The third sample showed some contraction of the base with most of the observations now falling in the 2V–4V range, while the second sample took an intermediate place between these two extremes. Henceforward differences in the distribution curves followed the pattern set in the first generation except that the final sample approximated more closely to the first.

A pattern of change in the sizes of the nuclei of the cells of an ascites tumour *in vivo* was thus established which again was not cumulative from one generation to the next.

FIG. 4. Distribution graphs of the relative sizes of the nuclei in generation G1 of tumour BAS/56AA at different stages in the life of the tumour.

4. *Sarcoma BAS/56AA*.—It was now decided to examine a tumour of entirely different type (BAS/56AA) and distribution curves were constructed as for the second generation of Epithelioma 255/Bl, again making 500 observations for each distribution curve.

The distribution curve of the first sample of BAS/56AA was again widely based and skewed to the right with the bulk of the observations in the 4V–8V range. The second sample did not differ markedly from the first. The curve for the third sample, however, showed marked contraction of the base, little skewing and the bulk of the observations in the 2V–4V range. Subsequent samples showed intermediate distributions between these two extremes until the curve for the final sample approximated closely to that for the first.

Thus, the general pattern of change in the sizes of the nuclei of the cells of Epithelioma 255/Bl was repeated in the benzopyrene-induced sarcoma BAS/56AA.

### DISCUSSION

From these results it is clear that a pattern of change occurs in the proportions of cells with nuclei of different sizes during the life of individual generations of two murine ascites tumours. In the case of Epithelioma 255/Bl it has been demonstrated that this pattern is approximately reproduced in successive transplant generations but is not cumulative from one generation to the next.

The distribution curves show that it is probable that the pattern of change cannot be attributed to variations in the rate of mitosis affecting the entire cell population equally. To produce the changes observed in the early life of the tumour would require a degree of synchrony of mitosis not usually seen in natural systems of adult mammalian cells ; similarly the changes cannot be explained on the basis of the addition and removal of nuclear constituents throughout the entire population. It appears that the explanation of the changes is to be sought in terms of differential changes within the population.

Increase in nuclear size in non-neoplastic tissues, other than that involved in reproductive growth, has been shown to be due to one of three causes : development of polyploidy (e.g. Beams and King, 1942 ; Teir, 1944 ; Walker, 1958), a non-proportional increase in extra-chromosomal protein in certain hormone-regulated tissues and following the action of certain chemical substances (Laird, 1953 ; Alfert, Bern and Kahn, 1955 ; Alfert, 1958) and nuclear oedema in certain degenerating, dying epithelial cells (Bern, Alfert and Blair, 1957).

In tumours, Kit (1960) has reported a correlation between chromosome ploidy and *inter alia*, cell volume, nuclear volume and nucleic acid content of cells of carcinomas and lymphomas ; Klein (1951) notes a " rough correlation " between cellular or nuclear size and the mean values of DNA per cell in a number of ascites tumours, and Hauschka (1961) a direct quantitative relationship between chromosome number and the parameters of DNA, cell volume and respiration. Richards and Davies (unpublished data cited by McLeish, 1960) find that, in the cells of a murine ascites tumour, the total amount of nuclear protein doubles during interphase but that its average increase, unlike that of DNA, is approximately linear.

The results of the experiments here reported are consistent with a heterologous tumour-cell population reacting to varying environmental pressures which favour the accumulation, by growth advantage gained, or by superior capacity for survival, of one or another of the cell types in the population ; if the correlations reported by Kit, by Klein and by Hauschka obtain under the conditions of these experiments, it would be proper to interpret these results as examples of genotype selection by varying environmental conditions. Experiments have been planned to test this interpretation.

### SUMMARY

1. A pattern of change is reported in the proportions of cells with nuclei of various sizes in a strain-specific ascites subline of a naturally-occurring murine epithelioma *in vivo*, under conditions where the environment varies during the course of an experiment, but which at any one time is the same for all the cells under test.

2. This pattern of change is approximately repeated in successive transplant generations of the tumour, but is not cumulative from one transplant generation to the next.

3. The pattern of change in the epithelioma is repeated in a strain-specific benzopyrene-induced murine sarcoma in similar conditions.

4. Mechanisms whereby the changes may have been brought about are briefly discussed.

The author is indebted to Dr. H. B. Fell, F.R.S., both for her encouragement of this work and for her help in the presentation of the results. Thanks are also due to Dr. D. J. Trevan for supplies of tumour Epithelioma 255/Bl. Technical assistance was provided by Miss V. Cable.

### REFERENCES

ALFERT, M.—(1958) *Exp. Cell Res.*, Suppl. **6**, 277.
*Idem*, BERN, H. A. AND KAHN, R. H.—(1955) *Acta anat.*, **23**, 185.
BEAMS, H. W. AND KING, R. L.—(1942) *Anat. Rec.*, **83**, 281.
BERN, H. A., ALFERT, M. AND BLAIR, S. M.—(1957) *J. Histochem. Cytochem.*, **5**, 105.
FOULDS, L.—(1951) *Ann. R. Coll. Surg. Engl.*, **9**, 93.
HAUSCHKA, T. S.—(1961) *Cancer Res.*, **21**, 957.
KIT, S.—(1960) *Ibid.*, **20**, 1121.
KLEIN, G.—(1951) *Exp. Cell Res.*, **2**, 518.—(1961) " Biological Approaches to Cancer Chemotherapy ", Edited by Harris, New York (Academic Press), p. 201.
LAIRD, A. K.—(1953) *Arch. Biochem. Biophys.*, **46**, 119.
MCLEISH, J.—(1960) " The Cell Nucleus ", London (Butterworth), p. 91.
ROBERTS, D. C. AND TREVAN, D. J.—(1960) *Brit. J. Cancer*, 14, 716.
TEIR, H.—(1944) *Acta path. microbiol. scand.*, Suppl. 56.
TREVAN D. J. AND ROBERTS, D. C.—(1960) *Brit. J. Cancer*, **14**, 724.
WALKER, B. E.—(1958) *Chromosoma*, **9**, 105.